# Tension pneumoperitoneum: Case report of a rare form of acute abdominal compartment syndrome

**DOI:** 10.1016/j.ijscr.2019.01.014

**Published:** 2019-01-29

**Authors:** Manuel Cadena, Jaime Solano, Nicolas Caycedo, Daniel Gomez, Eric E. Vinck, Pedro Quiroga, Paula Gaete

**Affiliations:** a Department of Surgery, Fundación Santa Fé de Bogotá, Bogotá, Colombia; b Universidad de Los Andes, Colombia; c Department of Surgery, Universidad El Bosque, Bogotá, Colombia; d Dr. Horacio Oduber Hospital, Oranjestad, Aruba; e Department of Medicine, Universidad de Los Andes, Bogotá, Colombia

**Keywords:** Abdominal compartment syndrome, Pneumoperitoneum, Tension pneumoperitoneum, Surgical decompression, Laparotomy

## Abstract

Introduction: Tension pneumoperitoneum is a severe and rare form of pneumoperitoneum with concomitant hemodynamic instability and respiratory failure. It is a variant of abdominal compartment syndrome (ACS) causing an abrupt increase in intra-abdominal pressure.

Presentation of case: We present a case of pneumoperitoneum, after an endoscopic mucosal resection with the development of ACS. The patient was successfully treated with percutaneous decompression.

Discussion: Decompressive laparotomy is the first treatment option for both most forms of pneumoperitoneum and ACS; nevertheless, this issue is controversial. Recent reports have shown that some patients may be candidates for a minimally invasive catheter decompression avoiding major decompressive surgery. Identifying these patients is vital to avoiding unnecessary surgeries.

Conclusions: Tension pneumoperitoneum is a life-threatening event, early detection and intervention is critical in order to provide prompt and optimal treatment approaches.

## Highlights

• Pneumoperitoneum is a rare cause of ACS.
• A defined approach has not been established.
• Whenever possible a minimally invasive approach should be attempted.
• Avoiding laparotomy may benefit certain patients.
• Reports are important in order to establish a treatment protocol.

## 1 Background

Pneumoperitoneum is defined as the presence of extraluminal air in the abdominal cavity [1]. The etiology of this condition is divided into two categories: the surgical pneumoperitoneum due to a perforation of a hollow organ (80–90% of cases), and the nonsurgical or spontaneous pneumoperitoneum (10–20% of cases), with ventilator induced barotrauma as the leading underlying condition [1]. The former, generally, requires prompt surgical exploration and intervention [2], while the latter is associated with therapeutic dilemma. Additionally, in the medical literature, tension pneumoperitoneum denotes an extreme form of pneumoperitoneum in which the elevated intra-abdominal pressure (IAP) causes hemodynamic instability and respiratory failure [3,4]. Abdominal volume expansion due to air into the peritoneal cavity increase the IAP and can lead to intra-abdominal hypertension (IAH) or abdominal compartment syndrome (ACS). The World Society of the Abdominal Compartment Syndrome (WSACS) defines IAH as a sustained or repeated pathological elevation in IAP ≥ 12 mmHg and ACS as a sustained IAP > 25 mmHg associated with new organ dysfunction/failure [5]. Though IAH and ACS are common in ICU patients (21–87% for IAH and 1–12% for ACS), these pathologies remain highly misunderstood and unrecognized [6,7]. Therefore, both IAH and ACS are responsible for a high burden on morbidity and mortality in critically ill patients [6]. To seed the suspicion of IAH and ACS in surgeons, anesthesiologists, intensivists and general practitioners; we present a case that highlights the burden, clinical presentation and spectrum of etiologies of elevated IAP syndromes. In addition, we discuss definitions and review similar cases. This case report was written following SCARE guidelines [8].

## 2 Case report

**Fig. 1:**
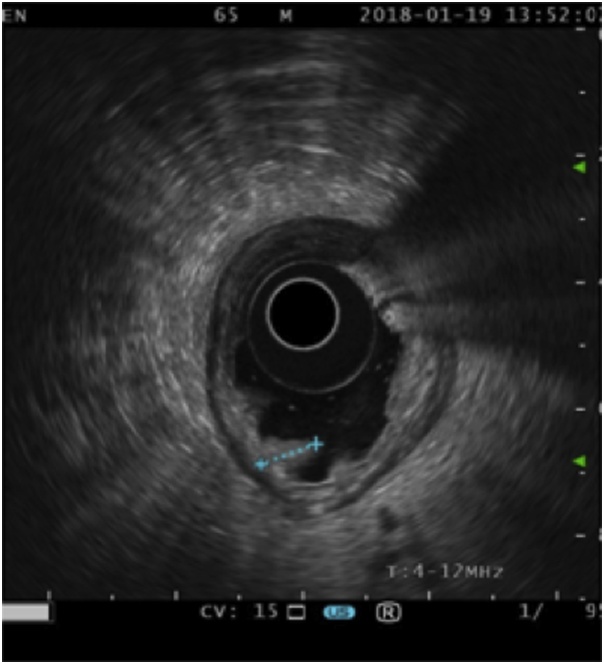
Endoscopic ultrasound showing a 32 mm adenocarcinoma (dots). The ulcerated tumor does not invade the muscularis.

**Fig. 2:**
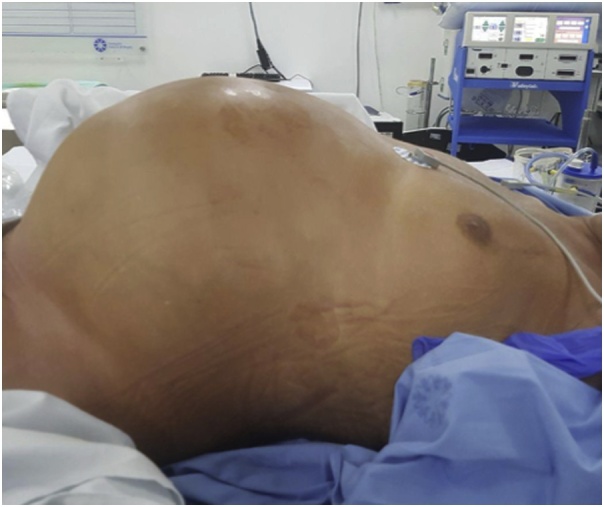
Distended abdomen at the end of the endoscopic procedure.

**Fig. 3:**
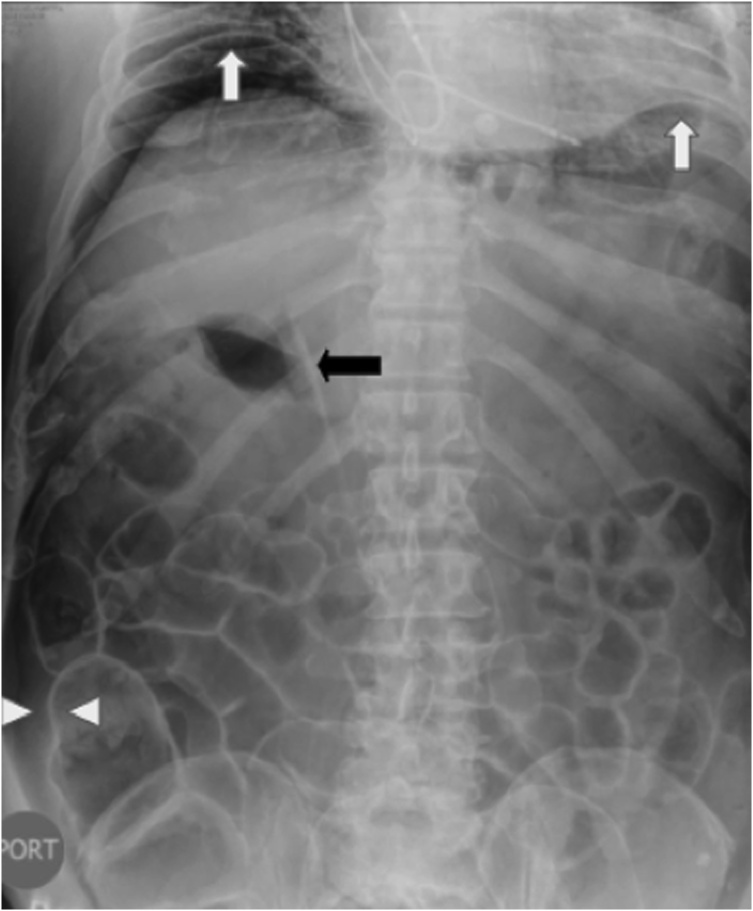
Abdominal radiography showing sub-diaphragmatic free air (white arrows), Rigler´s sign (white triangles), air in both sides of the intestine wall; and Falciform ligament sign (black arrow).

**Fig. 4:**
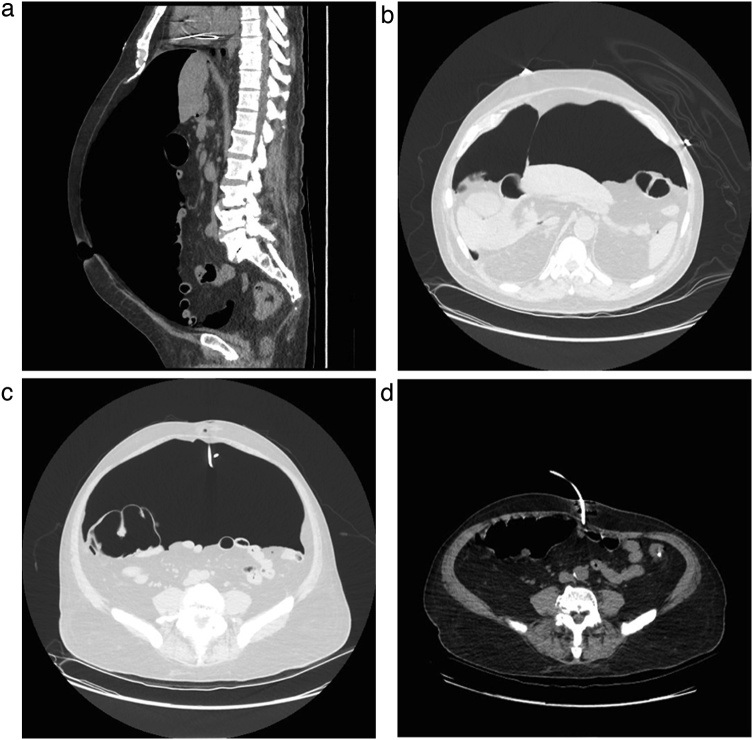
Abdominal CT-scan. **a.** Sagittal view showing the presence of air in the peritoneal cavity with posterior displacement of the intra-abdominal contents **b.** Axial view, showing the Falciform ligament sign (white arrow). **c.** Axial view showing percutaneous decompression with a multipurpose drainage catheter (white arrow) in the periumbilical position. **d.** Axial view after percutaneous decompression.

A 65-year old male patient with early gastric cancer was transferred from Aruba to our institution. He had a 3-year history of black stools and anemia. His past medical history included multiple comorbidities: diabetes, chronic renal failure, alcoholic cirrhosis Child A, complete heart blockade and thrombocytopenia of unknown etiology. An upper endoscopy and biopsy revealed a well-differentiated intestinal type adenocarcinoma in the antrum. Endoscopic ultrasonography showed a hypoechoic, 3.2 cm neoplasm, without muscularis externa infiltration and reactive ganglia (Fig. 1). Endoscopic mucosal resection was chosen due to tumor size, stage and comorbidities of the patient. The tumor was fully resected without complications. At the end of the procedure the anesthesiologist had difficulty with ventilation and abdominal distention was observed (Fig. 2). He had a 128/91 mmHg blood pressure and 70 bpm heart rate. An endoscopic revision was done before finishing the procedure, without identification of any macroscopic perforation. A nasogastric tube was placed and therapeutic strategies to improve abdominal-wall compliance were instituted (changes in ventilation parameters, nasogastric suction, change to a supine position and removal of any strap over the abdomen). A plain abdominal radiography in the operating room showed a massive pneumoperitoneum (Fig. 3). Decision of a nonsurgical management was conducted and the patient was taken to the intensive care unit (ICU) for monitoring. The IAP measured by a trans-bladder catheter was 33 mmHg. Six hours after ending the procedure the patient developed dyspnea and anuria. The diagnosis of an abdominal compartment syndrome was established. Given the worsening status, interventional radiology evaluated the patient. A CT scan confirmed the massive pneumoperitoneum without intraperitoneal extravasation of contrast (Fig. 4a & b). A percutaneous decompression guided by CT scan was performed with a pigtail catheter G14 (Fig. 4c & d). Air was immediately released under pressure. Immediately after the procedure, the patient's symptoms and hemodynamic status improved. Diuresis returned after a few hours. The pigtail catheter was closed the first day after placement and taken out at the third postoperative day. Control CT scan revealed no evidence of pneumoperitoneum. Pathology report confirmed the resected specimen had free malignant cell margins and areas of high-grade and low-grade dysplasia. The patient was discharged from ICU at postoperative day 2 and discharged from hospital at postoperative day 5 without further complications.

## 3 Discussion

We present a case of abdominal compartment syndrome (ACS) induced by pneumoperitoneum after an endoscopic mucosal resection. The WSACS defines ACS as IAP > 20 mmHg and new organ dysfunction/failure [5]. ACS is classified according to the origin of the disease: primary ACS when etiology is located into the abdominopelvic region and secondary ACS if the etiology is located outside the abdominopelvic region [5]. ACS is common in critically ill patients [7]. Several risk factors for the development of IAH/ACS have been described and are extensively reviewed in the last guideline from the WSACS [5]. In the present case, the main risk factor is the endoscopic procedure with air for a 2-h period. Microperforation can make a unidirectional valve in which air come out of the gastrointestinal tract and into de abdominal cavity. Similarly, according to risk factors, a broad range of substances increases the volume and pressure into the abdomen: air, tissue edema, liquid such as ascites and blood, or solids such as a tumor or gravid uterus [7]. In the same way, pneumoperitoneum (PP) has a wide spectrum of etiologies. Mularski et al. conducted a systematic review of nonsurgical causes of PP from 1970 to 1999; they classified this entity into four categories: iatrogenic, spontaneous, traumatic and miscellaneous [[9], [10], [11], [12]]. Iatrogenic PP is the most frequent category. They report that the occurrence of pneumoperitoneum after percutaneous endoscopic gastrostomy, diagnostic and therapeutic colonoscopy is up to 25%, 1% and 3% respectively. Additionally, in 85% to 95% of cases, PP reflects visceral perforation [[9], [10], [11], [12]]. In this case, neither endoscopic revision of exposed muscularis propria nor CT scan revealed an evident perforation that could benefit from an endoscopy or open treatment. PP as a complication of endoscopic procedures should be suspected as rate of use and applications of endoscopy are continuously increasing.

Literature review of similar cases is summarized in Table 1. A total of N = 13 similar reports were found. The mean age was 64.5 and 61% (N = 8/13) of cases were older than 70-year old. The sex distributions were similar (54% female). As reported in the systematic review of PP by Mularski et al. the most frequent etiology of PP was iatrogenic (N = 7/13; 54%), followed by spontaneous (N = 3/13; 23%), miscellaneous (N = 2/13;15%) and traumatic (1N=/13; 8%) [[8], [9], [10], [11], [12], [13], [14]]. Multiple organ failure in those patients is common; 59% of patients had at least two affected organs. Cardiovascular and respiratory systems were the most frequently affected and the clinical presentation was highly variable (Table 2). Laparotomy predominated to treat ACS secondary to PP: laparotomy was used in 54% of patients, PCD with subsequent laparotomy in 23% and PCD alone in 23% of patients. Finally, outcomes were reported in N = 9 patients; N = 4 (45%) died and N = 5 (55%) were discharged from hospital. This finding suggests that ACS worsens mortality rate in patients with PP [[12], [13], [14], [15], [16]].

**Table 1:** Summary of 13 cases of tension pneumoperitoneum.

Variable	Events (n)	Percentage (%)
Included studies	13	100%
Age (mean ± SD)	645 ± 16,3	
Sex		
Female	7	54%
Etiologic classification		
Iatrogenic	7	54%
Spontaneous	3	23%
Trauma	1	8%
Miscellaneous	2	15%
Injury localization		
Unknown	5	36%
GIT	7	50%
Airway	2	14%
Treatment		
PCD	3	23%
OAD	7	54%
PCD and then OAD	3	23%
Response to treatment		
Immediate	10	77%
Not reported	3	23%
Outcome		
Hospital discharge	5	38%
Death	4	31%
Not reported	4	31%

GIT, gastrointestinal tract; OAD, open abdominal decompression; PCD, percutaneous decompression; SD, standard deviation.

**Table 2:** Clinical presentation of organ failure in 12 patients with ACS secondary to pneumoperitoneum.

Clinical finding	Events (n)	Percentage (%)
Organs affected		
1	5	42%
2	5	42%
3	2	17%
4	0	0%
CNS		
Altered mental status	1	8%
Cardiovascular		
Decreased MAP	4	33%
Low systolic or diastolic pressure	4	33%
Diminished distal perfusion	2	17%
Bradycardia	1	8%
Respiratory		
Hypoxemia	5	42%
Dispnea	2	17%
Respiratory failure	2	17%
Cianosis	1	8%
Renal		
Oliguria	4	33%
Unclassified		
Metabolic acidosis	1	8%

IAH and ACS were defined 25 years ago but they remain poorly understood and under recognized [[7], [8], [9], [10], [11], [12], [13], [14], [15], [16], [17], [18]]. This fact explains the lack of standardized definitions and the existence of different definitions to the same clinical entity. One of those definitions is tension pneumoperitoneum (TP). TP denotes a severe presentation of PP and is defined as intraperitoneal gas, under pressure, that causes hemodynamic and ventilator compromise, needing urgent intervention [[4], [5], [6], [7], [8]]. Several cases of ACS induced by PP are founded in medical literature with the term tension pneumoperitoneum. Those cases have varied etiologies like colonoscopic complications [[18], [19], [20], [21], [22]], metastasis-induced perforation [23], perforated gastric ulcer [24]; and severe consequences of aortic occlusion [25]. Rogers and Garcia stated: irrespective to the cause, elevated IAP can threaten perfusion and thus viability of tissue in the abdominal compartment [7]. IAH impairs venous return from the brain; diminish cardiac output, renal and bowel perfusion and lungs residual capacity; and increase ventilator pressures [26]. Considering the multiorganic effects of IAP and previous suggestions by Peppriell and Bacon [16], we consider that a better definition for tension pneumoperitoneum is: intraperitoneal gas, under pressure, that causes abdominal compartment syndrome, as defined by the WSACS.

WSACS recommends a step-by-step approach to treat IAH/ACS [5]. Treatment options are classified into nonsurgical and surgical therapies. Nonsurgical therapies are: 1) improvement of abdominal wall compliance, 2) evacuation of intra-luminal contents, 3) correction of fluid balance, 4) organ support and 5) evacuation of abdominal collections (e.g. percutaneous catheter decompression). Nonsurgical therapies are the first line to treat IAH. In contrast, open abdomen decompression (OAD) is indicated to manage primary and secondary ACS and IAH refractory to nonsurgical treatment [5]. Nevertheless, although potentially lifesaving, OAD has high morbidity, due to complications like enteric fistulae and chronic incisional hernia; and high mortality rates [7,20]. As this case underscores, percutaneous catheter decompression (PCD) is emerging as first-line treatment for ACS. In one study, Cheatham and Safcsak prospectively recorded clinical and laboratory variables of patients who were treated with either PCD or OAD [20]. To evaluate the efficiency of PCD, PCD patients were matched with those requiring OAD according to age, sex, mechanism of injury, APACHE and SAPS II scores. The proportion of patients with ACS or IAH were 71% and 23% in PCD group and 68% and 6% in OAD group, respectively. PCD resolved organ failure and avoided OAD in N = 25 (89%) patients and both PCD and OAD were effective improving physiological variables [20]. Similarly, Peng et al. retrospectively analyzed outcomes in patients with severe acute pancreatitis and ACS [27]. ACS was managed with PCD (N = 212) or OAD (N = 61). OAD group had a larger improvement in physiological variables after decompression; however, a lesser proportion of PCD patients required ICU treatment (63% vs 98%, p < 0.001) and the mean stay for those who required ICU care was shorter in PCD group compared to OAD group (14 days vs 21 days, p < 0.001). The mortality in the PCD group was significantly lower than that of the OAD group (19% vs 52%, p < 0.001). Although those studies give positive insights in favor of PCD, evidence still is poor and randomized controlled trials are needed to define if PCD is superior as first-line treatment for patients with ACS [28].

## 4 Conclusions

Pneumoperitoneum is a rare cause of abdominal compartment syndrome. However, as endoscopic procedures are increasing, a rise in the incidence of ACS secondary to pneumoperitoneum is expected. Additionally, the current definition of tension pneumoperitoneum is incomplete not including the whole spectrum of complications due to pressurized air into the abdominal cavity [26]. A better definition of tension pneumoperitoneum is ACS secondary to pneumoperitoneum. Further research is needed to define the first-line treatment of ACS. Percutaneous decompression may be an effective alternative to avoid the complications after an open abdominal decompression.

## Conflicts of interest

Authors declare no conflicts of interests.

## Sources of funding

No funding was provided.

## Ethical approval

This report was exempted from ethics committee because of its case report nature.

## Consent

Patient and family gave their written consent for publication and is available if requested.

## Author contribution

Dr Cadena – attending surgeon made the clinical assessment of the patient and clinical decisions.

Dr Solano – Performed the ultrasongraphic procedures.

Dr Caycedo – attending surgeon treated the patient during the entire process.

Dr Gomez – article design and writing, English correction.

Dr Vinck – long-term follow-up of the patient, article final version style correction and English modification.

Dr Quiroga – literature review.

Dr Gaete – literature review.

## Registration of research studies

This was not a human study or clinical trial.

## Guarantor

Dr. Cadena.

Dr Vinck.

## Provenance and peer review

Not commissioned, externally peer-reviewed

## Author statement

As the field of medicine keeps on expanding, we are constantly challenged as physicians to provide the best care possible using less and less invasive approaches. This case report is a perfect example of a life-threatening emergency treated with an unconventional approach with excellent results. We hope that our case presentation will help guide other physicians in managing similar cases.
